# Association between dried fruit intake and kidney function: research from univariate and multivariate Mendelian randomized studies

**DOI:** 10.3389/fnut.2024.1440896

**Published:** 2024-10-23

**Authors:** Yuhang Gao, Xinghai Yue, Wanchao Zhao, Fang Yuan

**Affiliations:** ^1^The First Clinical College, Liaoning University of Traditional Chinese Medicine, Shenyang, China; ^2^The Second Clinical College, Liaoning University of Traditional Chinese Medicine, Shenyang, China; ^3^Department of Nephropathy, The First Affiliated Hospital of Liaoning University of Traditional Chinese Medicine, Shenyang, China

**Keywords:** Mendelian randomization, dried fruit intake, kidney function, genome-wide association study, causal relationship

## Abstract

**Objectives:**

Observational studies have identified an association between dried fruit intake and kidney function. However, these studies have limitations such as vulnerability to confounders and reverse causality bias. Therefore, this study aimed to explore the potential causal relationship between dried fruit intake and kidney function.

**Methods:**

A two-sample Mendelian randomization (MR) study was conducted using a large-scale genome-wide association study dataset to investigate the causal relationship between dried fruit intake and kidney function markers (blood urea nitrogen (BUN), creatinine (CR), uric acid (UA), cystatin C (CyC), hematuria, microalbuminuria). The main analytical method was inverse variance weighting. In addition, we applied the MR Egger and weighted median to assess the robustness of the results. Finally, Multivariate Mendelian randomization (MVMR) was used to estimate the direct effect of dried fruit intake on kidney function markers.

**Results:**

The univariate MR analysis showed that increased dried fruit intake was associated with lower kidney function markers, including BUN (*β*: −0.171, 95% confidence interval (CI): −0.239 to −0.102, *p* = 1.063 × 10^−6^), CR (*β*: −0.205, 95% CI: −0.311 to −0.099, *p* = 1.455 × 10^−4^), UA (*β* = −0.317, 95% CI: −0.384 to −0.249, *p* = 4.439 × 10^−20^), and CysC (*β* = −0.323, 95% CI: −0.384 to −0.249, *p* = 1.074 × 10^−11^); however, it was unrelated to hematuria and microalbuminuria. Causality persisted after performing MVMR analysis; however, with the addition of alcohol consumption and smoking as exposure factors, the causality for UA (*β* = −0.296, 95% CI: −0.523 to −0.068, *p* = 1.094 × 10^−2^) and CysC (*β* = −0.238, 95% CI: −0.465 to −0.011, *p* = 4.024× 10^−2^) weakened, while the causality for BUN (*β* = −0.038, 95% CI: −0.215 to 0.138, *p* = 6.698 × 10^−1^) and CR (*β* = −0.038, 95% CI: −0.431 to 0.046, *p* = 1.347 × 10^−1^) disappeared.

**Conclusion:**

Increased dried fruit intake was associated with lower kidney function markers (BUN, CR, UA, and CysC) in the absence of smoking and alcohol consumption; however, the causal relationship between dried fruit intake and BUN and CR disappeared in the presence of smoking and alcohol consumption. These results provide a promising avenue for delaying the course of chronic kidney disease.

## 1 Introduction

Patients with chronic kidney failure are often required to undergo treatments such as dialysis or kidney transplantation, which poses a huge global health challenge (1). According to the 2020 World Health Organization Global Health Estimates, kidney disease is among the top 10 causes of death worldwide (2). In 2024, the Global Kidney Health Atlas by the International Society of Nephrology reported that the global incidence of kidney failure is approximately 146 per million population per year (1). Currently, the main tests for kidney function include creatinine (CR), blood urea nitrogen (BUN), uric acid (UA), cystatin C (CysC), hematuria, microalbuminuria, etc., which represent the metabolic function and filtration function of the kidneys. Kidney disease progression is often associated with infections, inflammatory responses, and oxidative stress due to a weakened immune system throughout the body (3, 4). Furthermore, other acquired factors can also lead to progressive deterioration of kidney function markers, for example, hypertension, hyperlipidemia, and a hypercoagulable state (5). Current nutritional studies of kidney disease have identified an intrinsic relationship between dietary influences and the risk of progression of kidney function markers, which can be reduced by controlling the relevant factors (6).

The Mediterranean diet consists of a diet rich in vegetables, fruits, legumes, nuts, moderate amounts of poultry and seafood, very little red meat, and sweets (7). An article suggested that the Mediterranean dietary pattern improves lipid profiles in kidney transplant recipients and may help slow kidney failure in patients with chronic kidney disease (CKD) (8). Due to its healthy nutrient composition, it can benefit the intestinal microflora in various ways, thus ensuring the normal function of the intestinal barrier, in addition to having a beneficial effect on blood pressure (9–11). There is sufficient evidence to suggest that adherence to the Mediterranean diet is beneficial for patients with CKD, slowing the disease progression and reducing the likelihood of complications (12). Owing to the lack of preservation techniques in ancient times, a series of fruits, such as apricots and grapes, which are prone to decay, were often preserved by drying (13). Various previous studies have demonstrated that dried fruits have anti-inflammatory, antioxidant, and immune-boosting effects (14, 15); however, no cause-and-effect relationships have been documented, and there are no large-scale, multicenter, prospective studies to validate the benefits of dried fruit intake on the kidney function markers. Dried fruits are one dietary option available; however, many other dietary factors may have this effect.

Mendelian randomization (MR) employs genetic variation as an instrumental variable and capitalizes on the attributes of genetic variation to mitigate the impact of confounders and reverse causal bias.MR allows for the assessment of causal inference between exposure and outcome, opening new directions in the design and cost-effectiveness of clinical trials, and laying the groundwork for further research (16). MR of genetic variation as an instrumental variable analysis (IVs) avoids confounding factors and reverse causation on experimental results (17).

In this study, we focused on the causal relationship between dried fruit intake and kidney function markers (including CR, BUN, UA, CysC, hematuria, and microalbuminuria), to provide a scientific basis for delaying the course of CKD.

## 2 Materials and methods

### 2.1 Study design

**Figure 1:**
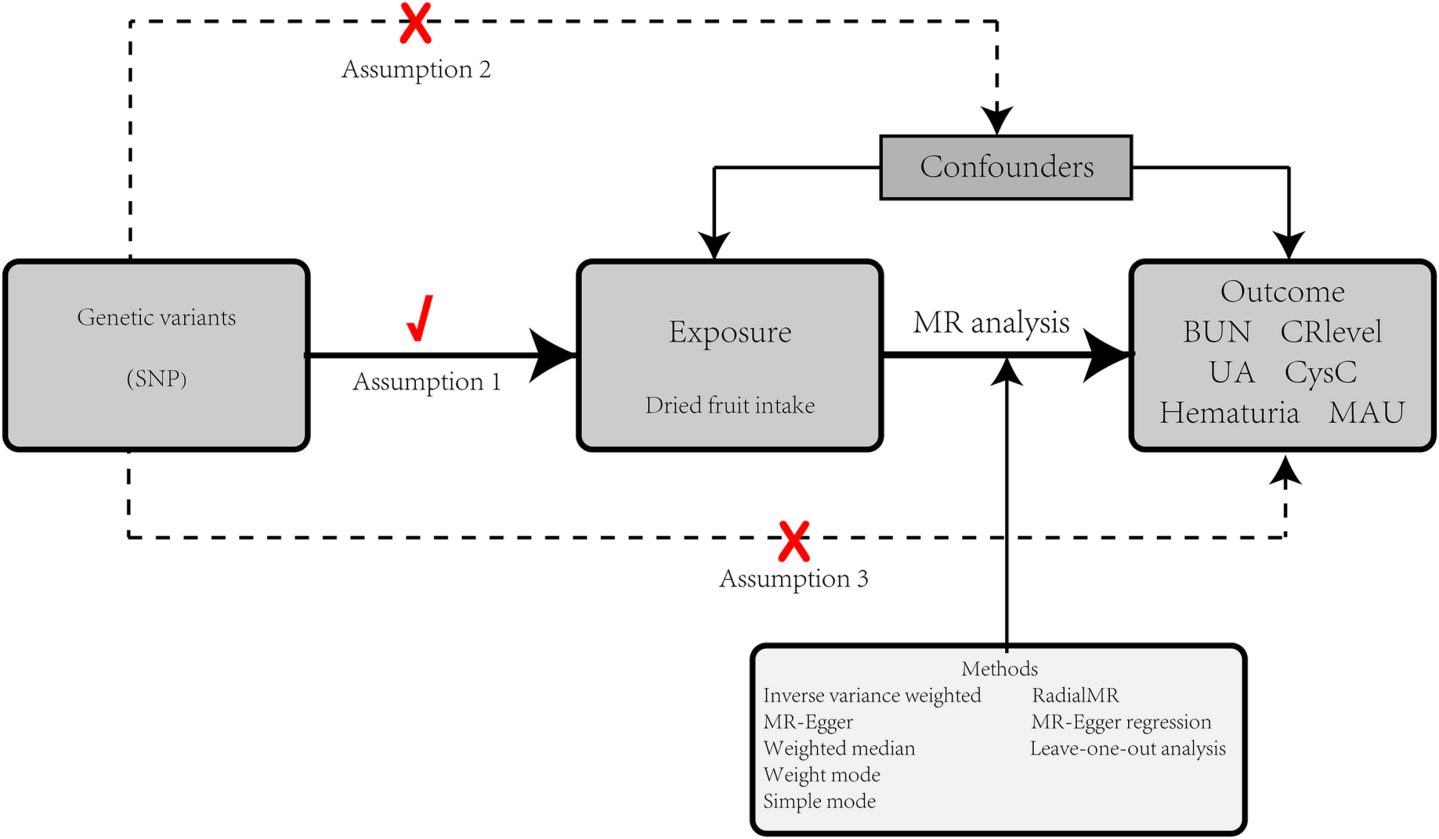
Study design.

We used two-sample MR analyses to investigate the relationship between dried fruit intake and kidney function markers. Multivariate Mendelian randomization (MVMR) was then used to analyze whether confounders would have an impact on the study results. Correct causal inferences are subject to the following conditions: (1) genetic instrumental variables should be highly correlated with exposure factors; (2) IVs are not associated with any possible confounders; and (3) IVs can influence the results only through exposure factors (16). The GWAS data involved in this study has been ethically approved (18–22). The study design is shown in Figure 1.

### 2.2 Data sources

The data involved in this study were obtained from the open GWAS database.^1^ Dried fruit intake was measured by a questionnaire entitled: “About how many pieces of dried fruit would you eat per day?” (count one prune, one dried apricot, and 10 raisins as one piece; if you do not eat any raisins, write “0”). The following check was performed during the collection process: if the answer was >100, the answer was invalid. Kidney function markers were derived from the following datasets for BUN, CR, UA, and CysC levels (UKB data field 30,720), diagnoses—main ICD10: R31 unspecified hematuria, and microalbuminuria. The MVMR analysis used fresh fruit intake (23), cooked vegetable intake (24), smoking status: current (25), and alcohol intake frequency (26) as confounders to further validate the relationship between dried fruit intake and kidney function markers. The specifics of the dataset are detailed in Table 1.

**Table 1:** Detailed information on included traits in the present study.

Phenotype	Consortium	Number of SNPs	Sample size	Ancestry	GWAS ID
Dried fruit intake	MRC-IEU	9,851,867	421,764	European	ukb-b-16576
Blood urea nitrogen levels	NA	19,049,084	344,052	European	ebi-a-GCST90018948
Creatinine levels	NA	11,590,399	110,051	European	ebi-a-GCST90092815
Serum uric acid levels	NA	19,041,286	343,836	European	ebi-a-GCST90018977
Cystatin C levels (UKB data field 30,720)	NA	10,783,683	389,834	European	ebi-a-GCST90014003
Diagnoses - main ICD10: R31 unspecified hematuria	MRC-IEU	9,851,867	463,010	European	ukb-b-17885
Microalbuminuria	CKD Gen	2,191,461	54,116	European	ieu-a-1097
Fresh fruit intake	MRC-IEU	9,851,867	446,462	European	ukb-b-3881
Cooked vegetable intake	MRC-IEU	9,851,867	448,651	European	ukb-b-8089
Alcohol intake frequency	MRC-IEU	9,851,867	462,346	European	ukb-b-5779
Smoking status: current	NA	10,894,596	336,024	European	ukb-a-225

### 2.3 IV selection

All genetic IVs involved in this study have a threshold of significance (*p* < 5 × 10^−8^) with respect to dry fruit intake; possible linkage disequilibrium was removed (r^2^ < 0.001, kb = 10,000). In addition, we applied the *F*-value (F = β^2^/se^2^) to assess the strength of each IV (27), with *F* > 10 indicating no weak instrumental bias. We obtained 43 single nucleotide polymorphisms (SNPs). Data are detailed in Supplementary Table S1.

### 2.4 Statistical analysis

Our main analytical method was inverse variance weighting (IVW), which is characterized by regressions that do not take into account the presence of an intercept term and are fitted with the inverse of the ending variance as weights (28). The advantage of this method is that it has a strong causality detection capability, which can effectively reduce bias and improve the accuracy of estimation, and the method can summarize the effects of multiple loci when dealing with multiple SNPs, thus providing more comprehensive results. The MR-Egger method is similar to the IVW method, but its regression takes into account the presence of the intercept term (29). When the above analysis is used in the MR analysis, the weighted median (WM) is more accurate in some cases after the results are validated (30). The weighting model weighs the effect of each SNP for further analysis (31).

For multi-effect outliers, we applied the Radial MR test and removed outliers (32). For horizontal pleiotropy, we used the MR-Egger intercept test. Cochran’s Q test was used to identify heterogeneity in the IVW analysis. For directional pleiotropy, we used funnel plot assessment. To determine whether each individual SNP was accountable for causal associations, we applied the leave-one-out method. To completely remove any SNP associated with potential confounders, we utilized the LDlink website (^2^last accessed on May 21st, 2024) and searched for all rsID after examining the phenotypes to remove any possible confounders. Based on the dietary recommendations mentioned in the *Dietary Guidelines for Americans (DGA)*, *2020–2025*, and a Meta-analysis of Lifestyles of Patients with Chronic Kidney Disease (33, 34). We selected several indicators, including fruit intake, cooked vegetable intake, smoking status: current, and frequency of alcohol intake. We selected several indicators, including fruit intake, cooked vegetable intake, smoking status: current, and alcohol intake frequency, and utilized MVMR analyses to further explore the effects of dried fruit intake on kidney function markers. All of the above analyses were statistically analyzed using R software (4.3.1) via “Two Sample MR” (version 0.5.11) and the “Radial MR” (version 1.0) (32) packages.

## 3 Results

### 3.1 Univariate MR results

A total of 43 SNPs were analyzed as IVs for MR in this study. We applied the Radial MR test to identify multi-effect outliers, and the results are detailed in Supplementary Figure S1.

After IVW, the results showed that a lower BUN (*p* = 1.063 × 10^−6^), CR level (*p* = 1.455 × 10^−4^), UA (*p* = 4.439 × 10^−20^), and CysC (*p* = 1.074 × 10^−11^) had a statistically significant association with dried fruit intake, with no association found for hematuria (*p* = 1.397 × 10^−1^) or microalbuminuria (*p* = 5.128 × 10^−1^; Supplementary Table S2; Supplementary Figures S2–S7).

**Figure 2:**
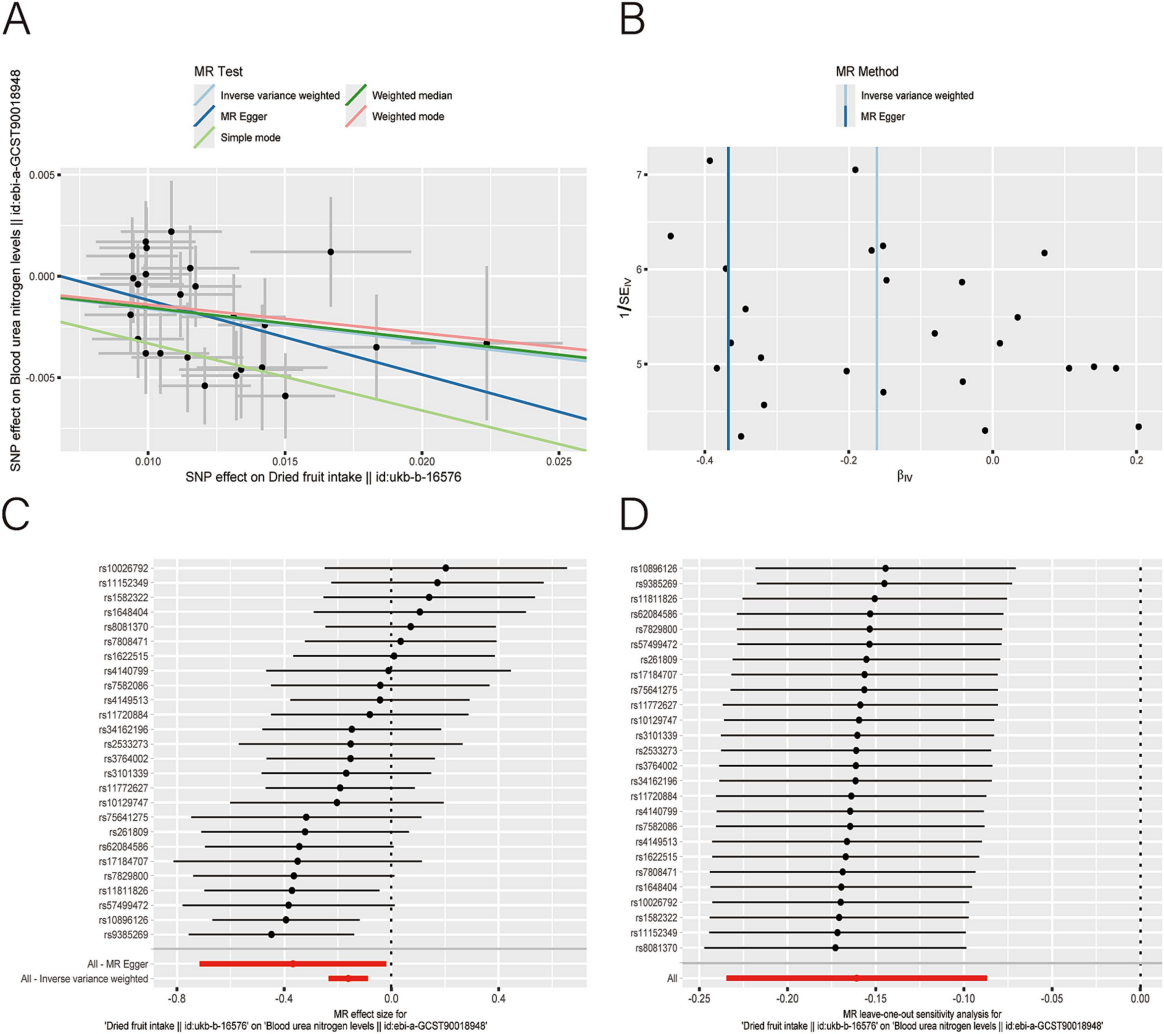
Scatter plot **(A)**, funnel plot **(B)**, forest plot **(C)**, and leave-one-out analysis **(D)** of single nucleotide polymorphisms (SNPs) associated with dried fruit intake based on blood urea nitrogen (BUN) level.

**Figure 3:**
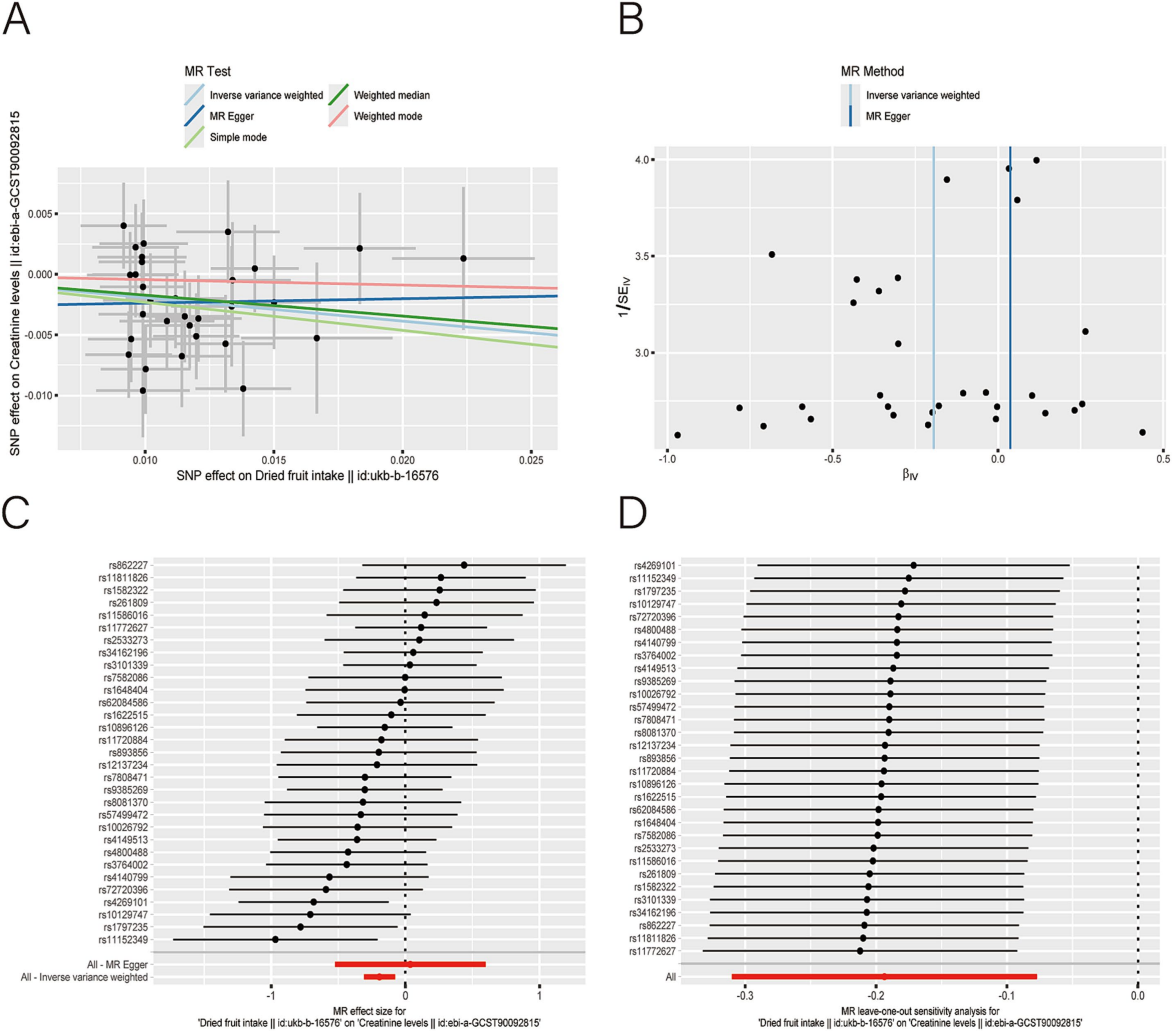
Scatter plot **(A)**, funnel plot **(B)**, forest plot **(C)**, and leave-one-out analysis **(D)** of single nucleotide polymorphisms (SNPs) associated with dried fruit intake based on serum creatinine (CR) level.

**Figure 4:**
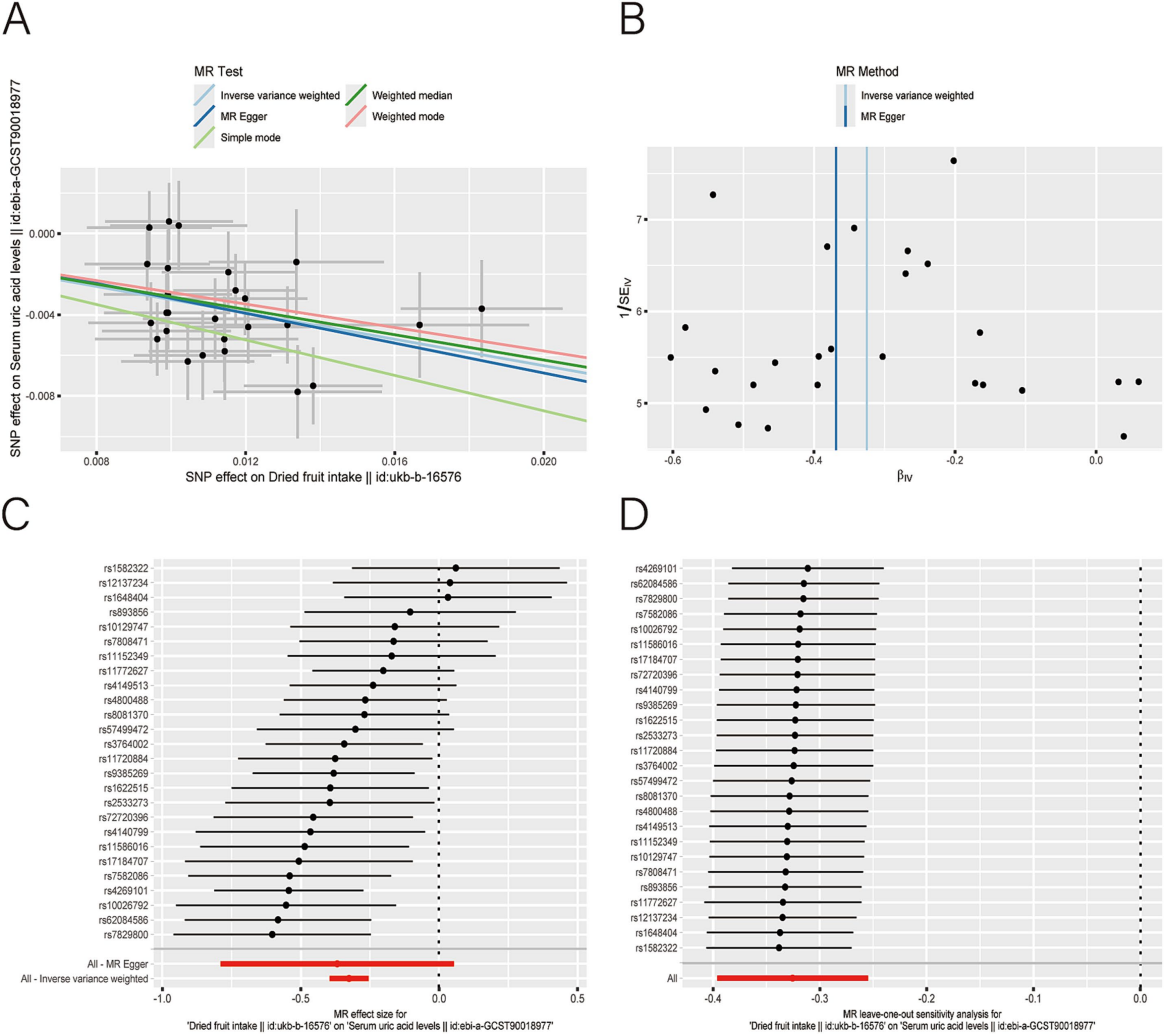
Scatter plot **(A)**, funnel plot **(B)**, forest plot **(C)**, and leave-one-out analysis **(D)** of single nucleotide polymorphisms (SNPs) associated with dried fruit intake based on serum uric acid (UA) level.

**Figure 5:**
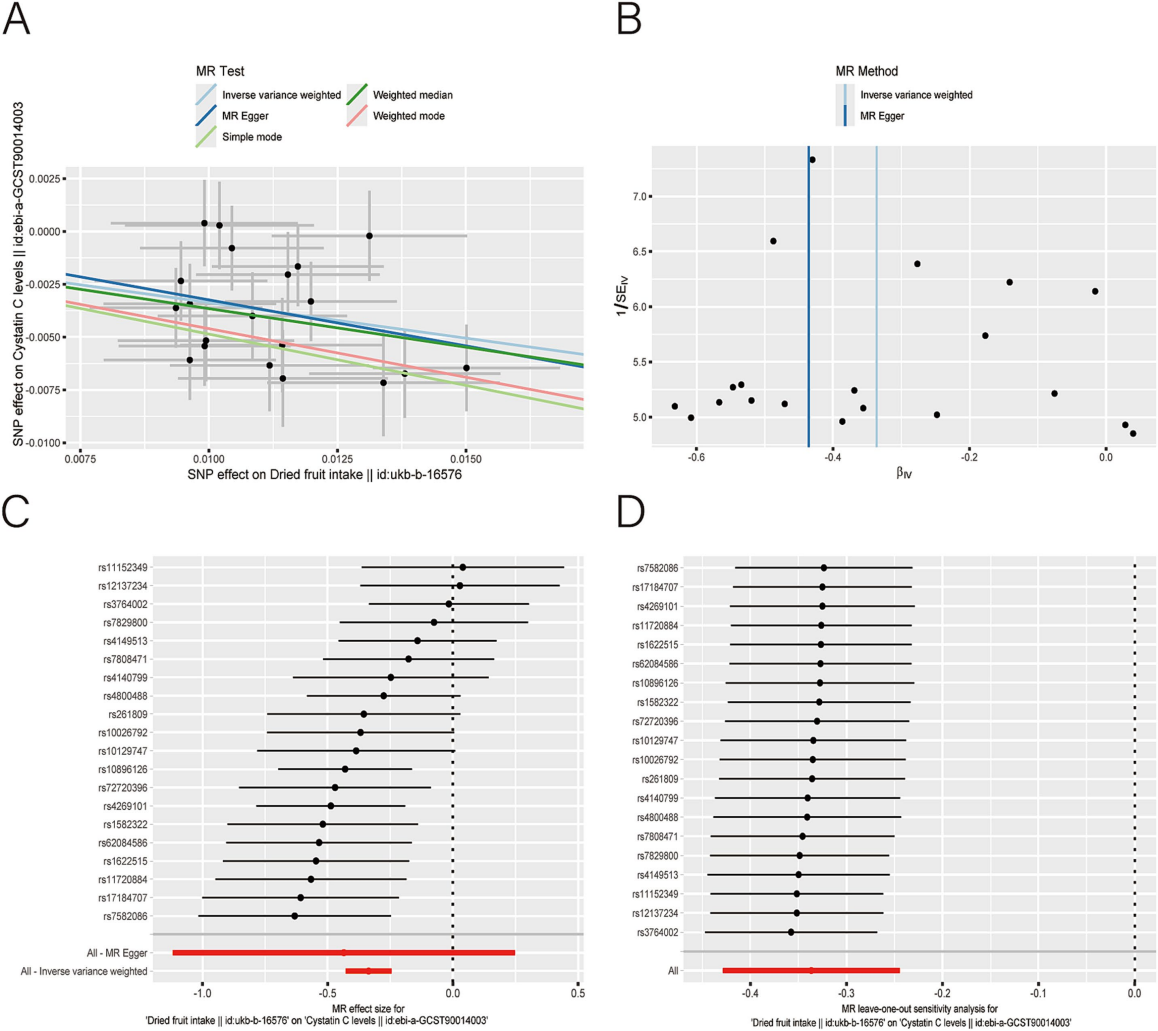
Scatter plot **(A)**, funnel plot **(B)**, forest plot **(C)**, and leave-one-out analysis **(D)** of single nucleotide polymorphisms (SNPs) associated with dried fruit intake based on cystatin C (CysC) level.

After applying LDlink (last accessed on May 21, 2024) to remove SNPs associated with other shapes (Supplementary Table S3), we found that dried fruit intake was associated with lower levels of BUN (*β*: −0.161, 95% CI: −0.235 to −0.087, *p* = 2.010 × 10^−5^), CR level (*β*: −0.194, 95% CI: −0.310 to −0.077, *p* = 1.135 × 10^−3^), UA (*β*: −0.326, 95% CI: −0.396 to −0.255, *p* = 1.894 × 10^−19^), and CysC (*β*: −0.337, 95% CI: −0.429 to −0.244, *p* = 8.227 × 10^−13^) (Table 2). The results were not heterogeneous or horizontally pleiotropic (*p* > 0.05; Supplementary Table S4). The funnel plot results show that the pattern is essentially symmetrical, proving to be unaffected by potential bias. The leave-one-out plot indicates that the result is not affected by individual variations (Figures 2–5).

**Table 2:** MR estimates of the causal association between dried fruit intake on BUN, CR, UA, and CysC level.

Exposure	Methods	BUN		CR level		UA		CysC	
		*β* (95% CI)	*p* value	*β* (95% CI)	*p* value	*β* (95% CI)	*p* value	*β* (95% CI)	*p* value
Dried fruit intake	Inverse variance weighted	-0.161 (−0.235 to −0.087)	< 0.001	-0.194 (−0.310 to −0.077)	< 0.05	-0.326 (−0.396 to −0.255)	< 0.001	-0.337 (−0.429 to −0.244)	< 0.001
	MR Egger	−0.367 (−0.716 to −0.019)	< 0.05	0.036 (−0.525 to −0.598)	> 0.05	−0.368 (−0.790 to 0.054)	< 0.05	−0.435 (−1.119 to 0.248)	> 0.05
	Weighted Median	−0.155 (−0.257 to −0.053)	< 0.05	−0.173 (−0.336 to −0.010)	< 0.05	−0.312 (−0.415 to −0.208)	< 0.001	−0.365 (−0.489 to −0.241)	< 0.05
	Weighted mode	−0.140 (−0.355 to −0.075)	> 0.05	−0.045 (−0.366 to 0.277)	> 0.05	−0.289 (−0.465 to −0.114)	< 0.05	−0.460 (−0.727 to −0.193)	< 0.05
	Simple mode	−0.331 (−0.565 to −0.097)	< 0.05	−0.232 (−0.594 to 0.131)	> 0.05	−0.437 (−0.649 to −0.225)	< 0.001	−0.486 (−0.759 to −0.212)	< 0.05

BUN, blood urea nitrogen levels; CR level, creatinine levels; UA, serum uric acid levels; CysC, cystatin C levels.

The relationship between dried fruit intake and renal physical and chemical tests was further analyzed using MVMR. When fresh fruit and cooked vegetable intake were used as covariates, there was still evidence of an association between dried fruit intake and lower BUN (*β*: −0.196, 95% CI: −0.364 to −0.027, *p* = 2.309 × 10^−2^), CR level (*β*: −0.258, 95% CI: −0.437 to −0.080, *p* = 4.494 × 10^−3^), UA (*β*: −0.452, 95% CI: −0.618 to −0.287, *p* = 8.987 × 10^−8^), and CysC (*β*: −0.393, 95% CI: −0.608 to −0.1178, *p* = 3.405 × 10^−4^). After adding smoking status: current to the covariates, the MVMR analysis was again performed and found that the MVMR estimates were weakened: BUN (*β*: −0.173, 95% CI: −0.345 to −0.002, *p* = 4.787 × 10^−2^), CR level (*β*: −0.266, 95% CI: −0.447 to −0.086, *p* = 3.760 × 10^−3^), UA (*β*: −0.421, 95% CI: −0.595 to −0.248, *p* = 1.961 × 10^−6^), and CysC (*β*: −0.347, 95% CI: −0.551 to −0.134, *p* = 8.400 × 10^−4^). After adding alcohol intake frequency to the covariates, the MVMR analysis was again performed and found that the MVMR estimates were attenuated or not significant: BUN (*β*: −0.038, 95% CI: −0.215 to −0.138, *p* = 6.698 × 10^−1^), CR level (*β*: −0.148, 95% CI: −0.341 to 0.046, *p* = 1.347 × 10^−1^), UA (*β*: −0.296, 95% CI: −0.523 to −0.068, *p* = 1.094 × 10^−2^), and CysC (*β*: −0.238, 95% CI: −0.465 to −0.011, *p* = 4.024 × 10^−2^; Table 3).

**Table 3:** Multivariate MR results of dried fruit intake with BUN, CR, UA, and CysC (IVW).

Exposure	BUN		CR		UA		CysC	
	*β*(95% CI)	*p* value	*β*(95% CI)	*p* value	*β*(95% CI)	*p* value	*β*(95% CI)	*p* value
Fresh fruit intake, cooked vegetable intake as covariates								
Dried fruit intake	−0.196(−0.364 to −0.027)	2.309 × 10^−2^	−0.258(−0.437 to −0.080)	4.494 × 10^−3^	−0.452(−0.618 to −0.287)	8.987 × 10^−8^	−0.393(−0.608 to −0.178)	3.405 × 10^−4^
Fresh fruit intake	−0.110(−0.319 to 0.099)	3.026 × 10^−1^	0.144(−0.074 to 0.362)	1.961 × 10^−1^	0.236(0.032 to 0.440)	2.323 × 10^−2^	0.139(−0.119 to 0.397)	2.898 × 10^−1^
Cooked vegetable intake	0.435(0.191–0.678)	4.784 × 10^−4^	−0.169(−0.428 to 0.091)	2.028 × 10^−1^	0.240(−0.002 to 0.482)	5.166 × 10^−2^	0.018(−0.302 to 0.338)	9.141 × 10^−1^
Added smoking status: current								
Dried fruit intake	−0.173(−0.345 to −0.002)	4.787 × 10^−2^	−0.266(−0.447 to −0.086)	3.760 × 10^−3^	−0.421(−0.595 to −0.248)	1.961 × 10^−6^	−0.347(−0.551 to −0.143)	8.400 × 10^−4^
Fresh fruit intake	−0.115(−0.320 to 0.091)	2.740 × 10^−1^	0.157(−0.057 to 0.371)	1.496 × 10^−1^	0.244(0.037 to 0.451)	2.089 × 10^−2^	0.153(−0.088 to 0.395)	2.138 × 10^−1^
Cooked vegetable intake	0.368(0.130 to 0.606)	2.454 × 10^−3^	−0.188(−0.439 to 0.063)	1.414 × 10^−1^	0.178(−0.065 to 0.422)	1.501 × 10^−1^	−0.028(−0.319 to 0.263)	8.518 × 10^−1^
Smoking status: current	−0.187(−0.644 to 0.270)	4.228 × 10^−1^	0.096(−0.375 to 0.566)	6.897 × 10^−1^	0.062(−0.403 to 0.527)	7.933 × 10^−1^	0.453(−0.065 to 0.971)	8.667 × 10^−1^
Added alcohol intake frequency								
Dried fruit intake	−0.038(−0.215 to 0.138)	6.698 × 10^−1^	−0.148(−0.341 to 0.046)	1.347 × 10^−1^	−0.296(−0.523 to −0.068)	1.094 × 10^−2^	−0.238(−0.465 to −0.011)	4.024 × 10^−2^
Fresh fruit intake	−0.250(−0.459 to −0.041)	1.906 × 10^−2^	0.004(−0.227 to 0.234)	9.752 × 10^−1^	0.040(−0.230 to 0.311)	7.695 × 10^−1^	0.179(−0.090 to 0.448)	1.917 × 10^−1^
Cooked vegetable intake	0.319(0.089 to 0.549)	6.639 × 10^−3^	−0.069(−0.320 to 0.181)	5.886 × 10^−1^	0.266(−0.032 to 0.563)	8.021 × 10^−2^	−0.226(−0.520 to 0.067)	1.302 × 10^−1^
Smoking status: current	−0.081(−0.524 to 0.361)	7.185 × 10^−1^	0.282(−0.190 to 0.754)	2.416 × 10^−1^	0.211(−0.361 to 0.783)	4.695 × 10^−1^	0.984(0.442 to 1.525)	3.690 × 10^−4^
Alcohol intake frequency	0.100(0.059 to 0.140)	1.293 × 10^−6^	0.085(0.034 to 0.136)	1.069 × 10^−3^	0.070(0.016 to 0.124)	1.105 × 10^−2^	0.092(0.032 to 0.152)	2.756 × 10^−3^

BUN, blood urea nitrogen level; CR, creatinine level; UA, serum uric acid level; CysC, cystatin C level.

## 4 Discussion

Our study showed that dried fruit intake has potential preventive value for increased BUN, CR, UA, and CysC levels, and no preventive value for hematuria and microalbuminuria. The results of the MVMR adjusted for fresh fruit intake, cooked vegetable intake, smoking status: current, and alcohol intake frequency found that in the absence of smoking and alcohol consumption as exposure factors, there was still substantial evidence that dried fruit intake was associated with BUN, CR, UA, and CysC levels, whereas when alcohol consumption was added as an exposure factor, the causality of dried fruit intake BUN and CR levels disappeared. The results suggest that this association may help to elucidate the pathophysiological mechanisms underlying the delayed effect of dried fruit intake on the course of CKD, which may be related to the prevention of an increase in kidney function markers. Thus, increased intake of dried fruits may help slow the deterioration of kidney function markers by reducing indicators of kidney function markers, although this effect is diminished or eliminated by smoking and alcohol consumption.

Dried fruit, as a preserved fruit substitute, ensured the supplementation of vitamins and other nutrients in ancient times when the preservation technology was not mature. Although the current development of preservation technology and transportation has ensured the freshness of fruits, their convenience, stability, and other characteristics are still widely popular. Currently, there are few studies on dried fruit intake for kidney disease, and people are more concerned about elements such as protein and energy (6). One of the characteristics of CKD is the increase in oxidative stress, which may be exacerbated by the gradual decrease in kidney excretory function and the accumulation of toxins as CKD worsens. Studies have shown that dried fruits are a rich source of antioxidant polyphenols; a study of common foods in the United States found that raisins (golden seedless) had the highest oxygen radical absorption capacity, followed by dried pears, dried plums, and so on (35). It has also been found that dried fruits always have higher antioxidant activity than the corresponding fresh dried fruits (36). Studies have demonstrated that the primary pathogenic mechanism of kidney injury is the inflammatory response (37). The prospective study “Systemic Inflammation Indicators Predict Survival in a U.S. CKD Population” (38) suggested that dried fruits are known to inhibit the inflammatory response. Furthermore, studies have shown that dried fruits can be used to prevent cancer (39) and reduce mortality (40). The theory of intestinal–kidney syndrome was presented by Ritz at the International Dialysis Congress in 2011; it states that damage to the intestinal barrier function can lead to pathology in several organs, including the heart and kidneys (41). In the same year, Meijers first introduced the concept of the gut–kidney axis and found that microbial levels in the gut can regulate uremic toxins in the body to influence the progression of CKD (42). In 2019 Meijers again interpreted the gut–kidney axis and found that CKD causes dysregulation of the gut microbiota, while ecological dysregulation of gut microbes can lead to the exacerbation of kidney disease (43). Dried fruits inherently contain many bioactive substances that can potentially influence gut microbial composition in ways that have potential health benefits (44).

BUN, CR, UA and CysC often represent the ability of the kidneys to excrete metabolic wastes in a clinical setting. Oxidative stress in kidney cells can lead to kidney damage, including damage to podocytes and the tubular interstitium of the kidney (45). Renal podocytes are an important part of maintaining the glomerular filtration barrier. Damage to podocytes can lead to kidney diseases such as proteinuria or glomerulosclerosis, and renal tubular mesenchyme plays an important role in maintaining renal function, which is closely related to the development of kidney diseases (46). As a portable grape product, Raisins are rich in nutrients, the most abundant of which are resveratrol and other phenols with anti-inflammatory and antioxidant properties (47, 48). Resveratrol has been found to reduce oxidative stress inflammatory cytokine levels, improve renal structure, and have a protective effect on the kidney (49). An *in vivo* experiment demonstrated that resveratrol inhibits and protects against podocyte apoptosis and improves kidney function (50, 51). A review of phenolic compounds shows that tannins and stilbenes contained in raisins can reduce the burden of oxidative stress on the kidneys and slow disease progression (52). Raisins were reported to reduce the risk of inflammatory injury through their effects on TNF-*α* (53). Black grapes were reported to improve the altered kidney function in hypercholesterolemic rats, and the effect is better than that of red grape juice (54). A review of dried plums showed that they were effective in promoting the growth of beneficial bacteria in the colon and in improving immune function (55). Furthermore, prunes, as a sub-species of plums, is also rich in phenolic compounds that inhibit the activation of the immune-inflammatory system and oxidative stress (56). Chlorogenic acid in dried plums was found to block inflammatory signaling pathways at multiple levels and reduce inflammatory damage (57). Apricots are a source of numerous vitamins and essential nutrients (58), and are often used as an antipyretic, laxative, emetic, etc. (59); they are rich in phenolic acids and flavanols, which reduce oxidative stress and regulate the balance of intestinal flora (14, 60–62). Japanese apricots (processed into umeboshi from dried and pickled forms) have been found to reduce gastrointestinal mucosal inflammation (63) and to improve the composition of the intestinal flora, with an increase in *Mycobacterium* spp. and *Clostridium* spp. cluster IV (64). Studies have found that apigenin, which is found in dried apricots, has a renoprotective effect and can reduce markers of renal function (65). However, there are still few studies on the association between dried fruits and CKD; therefore, animal experiments and large-scale clinical trials are needed to verify the efficacy.

Despite the numerous health benefits of dried fruits, they are often susceptible to contamination by various toxigenic fungi during the entire production process due to the specific way in which they are prepared. The most notable contaminants are aflatoxins (AFs) and ochratoxin A (OTA), with AFs leading to DNA mutations as well as liver and kidney damage, and OTA mainly leading to kidney damage (66). Therefore, care should be taken to purchase dried fruits from regular merchants to prevent damage.

Our MR results showed that there was a statistically significant association between dried fruit intake and lower BUN (*p* = 1.063 × 10^−6^), CR level (*p* = 1.455 × 10^−4^), UA (*p* = 4.439 × 10^−20^), and CysC (*p* = 1.074 × 10^−11^). In contrast, there was no statistically significant effect level for hematuria (*p* = 0.140) and microalbuminuria (*p* = 0.513). CR, BUN, UA, and CysC levels are the most commonly used indicators for assessing kidney function in patients with CKD, and they are considered to be closely related to the kidney’s ability to excrete metabolic waste from the body; therefore, they are easily influenced by dietary factors. Our results suggest that dried fruit intake has a causal effect on BUN, CR levels, UA, and CysC levels, which are influenced by smoking and drinking. More research is needed to confirm that dried fruit intake reduces CR, BUN, UA, and CysC levels in clinical trials.

There are some limitations to our study. First, MR relies on the validity of the GWAS results and is ethnically homogenous, limiting the ability to generalize the results. Second, possible confounding factors were not completely excluded from this study. Third, the dataset used in this study took the form of a questionnaire; the disadvantage of this method is that it may have incurred bias in the answers, thus affecting the accuracy. In conclusion, further *in vivo*, *in vitro*, and clinical trials are needed to provide more conclusive evidence. For example, studying the benefits of substances contained in dried fruits on kidney cells, or whether the addition of dried fruits intake to the daily diet of rats with chronic kidney disease could have a beneficial effect on the rate of kidney function markers such as BUN, CR, UA and CysC.

## 5 Conclusion

In conclusion, using a large-scale GWAS analysis of a European population, our study demonstrates that dried fruit intake is associated with lower BUN, CR, UA, and CysC levels, and that some of the results are influenced by factors such as alcohol and smoking. However, further studies are required to prove the causal relationship, which includes studies with different ethnic groups, followed by a comprehensive assessment of the impact of experimental participants’ lifestyles, social determinants, environmental exposures, and physicochemical tests that may affect kidney function markers.

## Data Availability

The original contributions presented in the study are included in the article/Supplementary material, further inquiries can be directed to the corresponding author.

## Footnotes

^1^ https://gwas.mrcieu.ac.uk

^2^ https://ldlink.nih.gov/?tab=ldtrait
