# Supplementary material for: Association between dried fruit intake and kidney function: research from univariate and multivariate Mendelian randomized studies
Source: Front Nutr. 2024 Oct 23;11:1440896. doi: 10.3389/fnut.2024.1440896 (PMC11537924; doi:10.3389/fnut.2024.1440896)
Supplement: Supplementary file 2 [file Image_1.pdf]

Supplementary Material

Association Between Dried Fruit Intake and Kidney Function: Research from Univariate and Multivariate Mendelian Randomized Studies

Yuhang Gao[1]†, Xinghai Yue[2]†, Wanchao Zhao[3]†, Fang Yuan[3]*

*** Correspondence:** Fang Yuan: 17702433892@163.com

Supplementary Figures

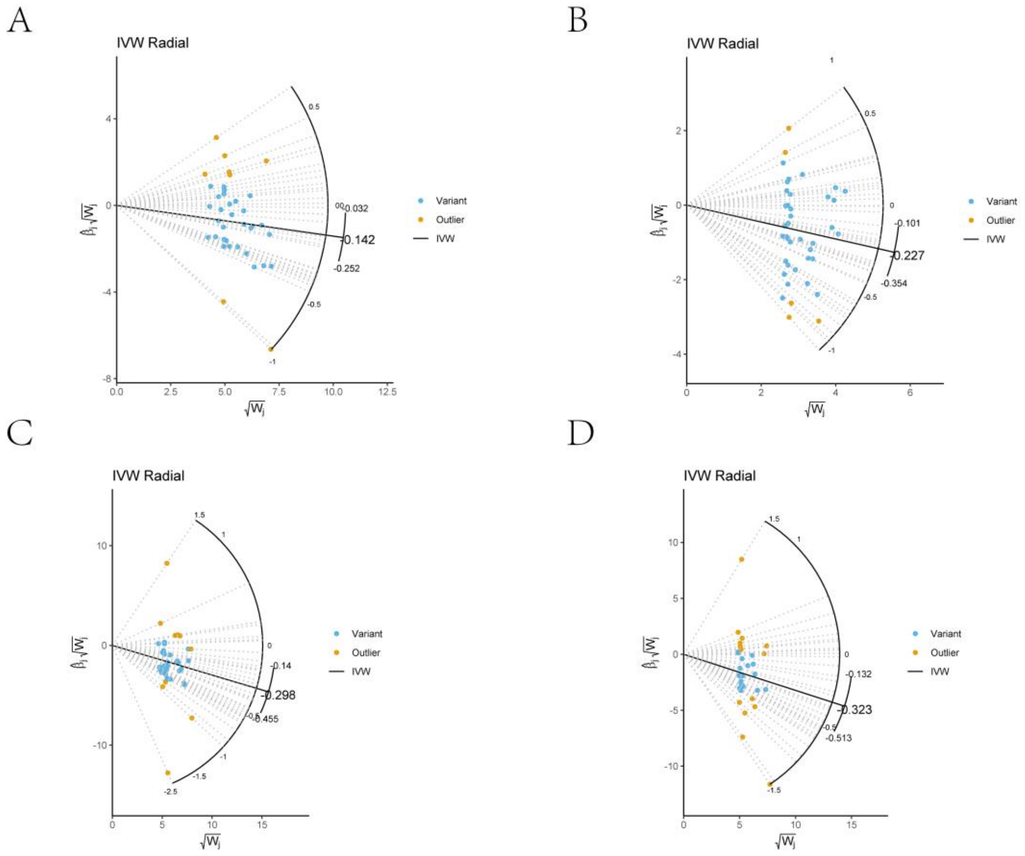

Supplementary Figure 1. Radial MR results of dried fruit intake on BUN, CR, UA, and CysC.

(A) Radial MR results of dried fruit intake on BUN.

(B) Radial MR results of dried fruit intake on CR.

(C) Radial MR results of dried fruit intake on UA.

(D) Radial MR results of dried fruit intake on CysC.

Abbreviations: BUN, Blood urea nitrogen**; CR,** Creatinine**; UA,** Uric acid**; CysC,** Cystatin C

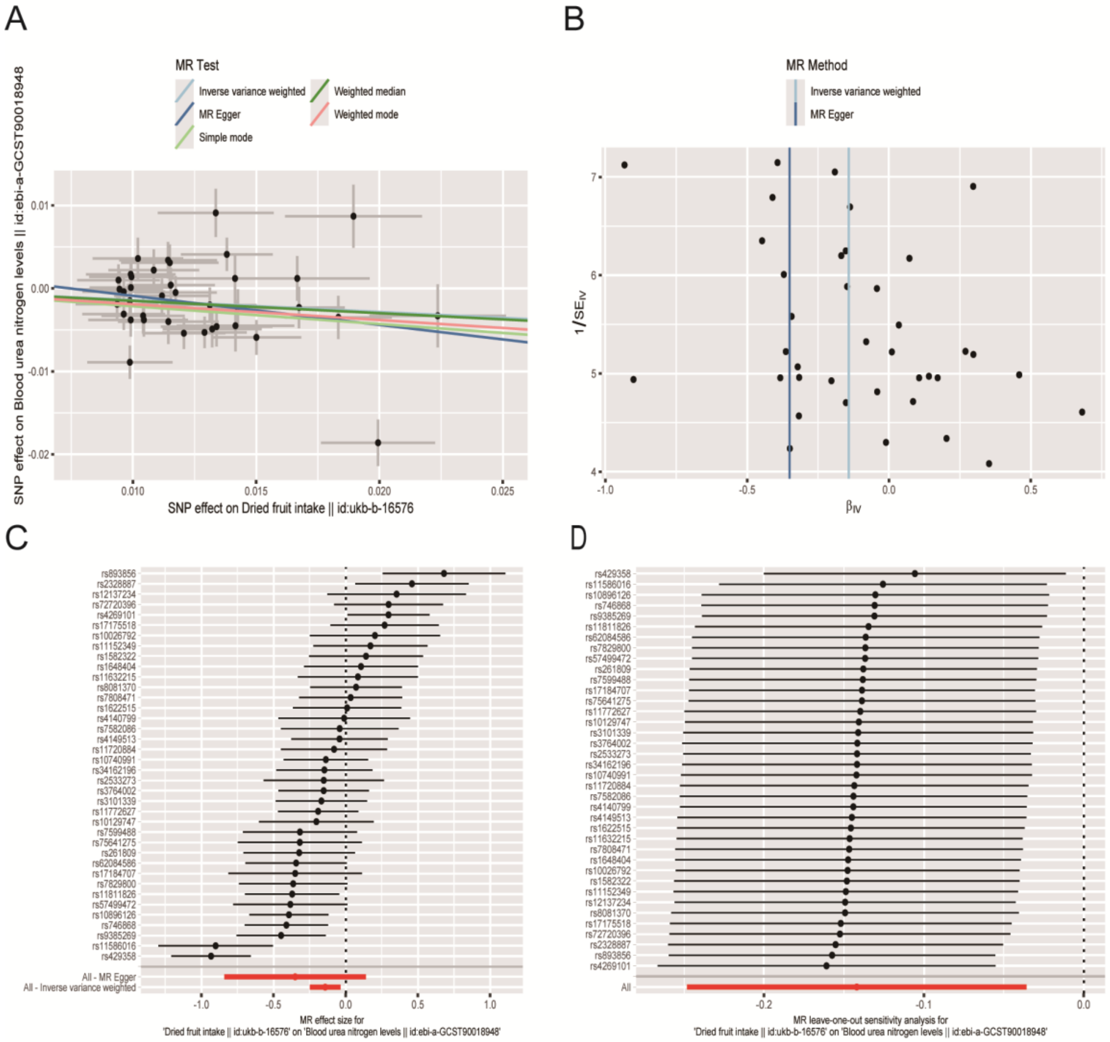

Supplementary Figure 2. MR results of dried fruit intake on BUN.

(A) Scatter plot of SNPs associated with dried fruit intake on BUN.

(B) Funnel plot of SNPs associated with dried fruit intake on BUN.

(C) Forest plot of SNPs associated with dried fruit intake on BUN.

(D) Leave-one-out analysis of SNPs associated with dried fruit intake on BUN.

Abbreviations: BUN, Blood urea nitrogen**; SNP,** Single nucleotide polymorphism

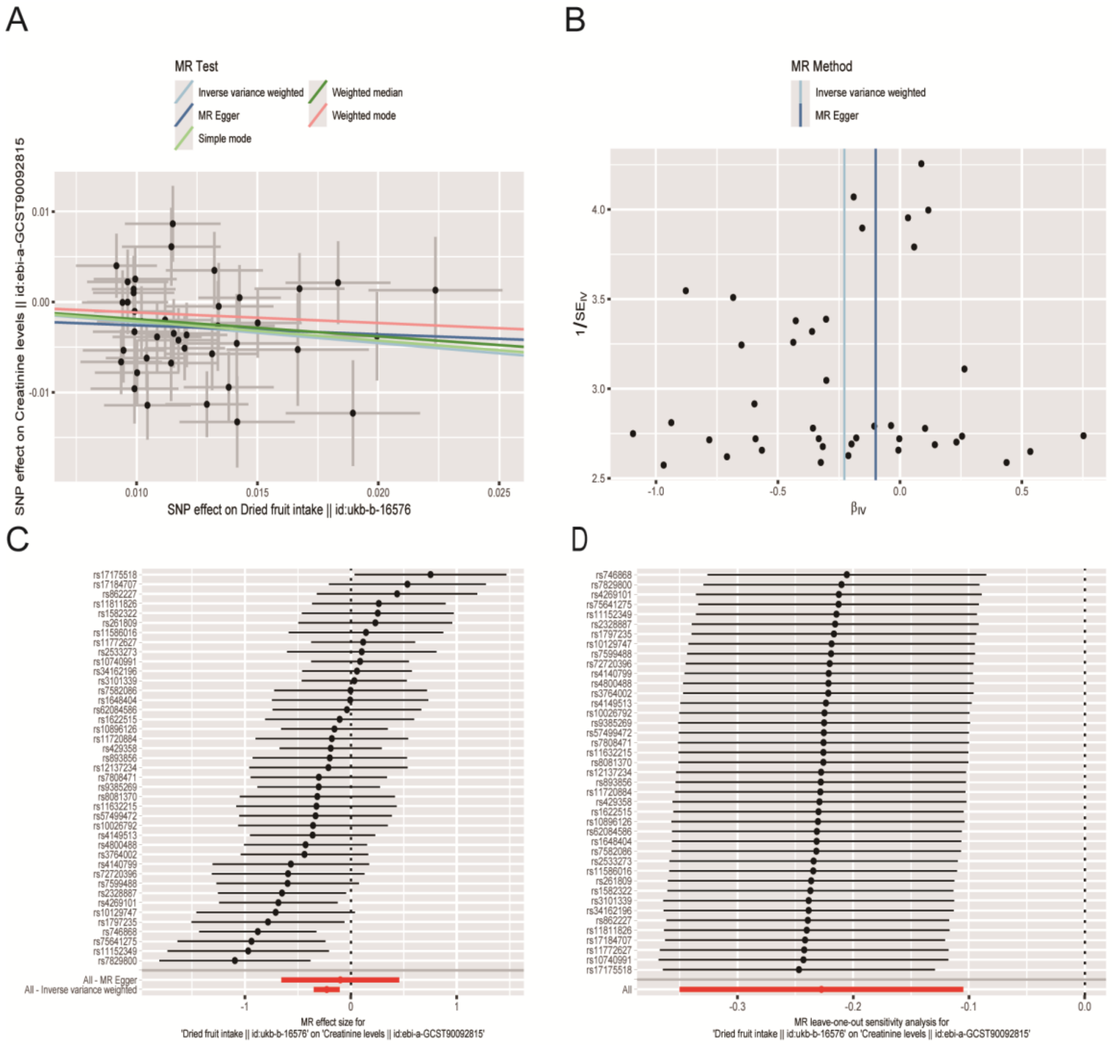

Supplementary Figure 3. MR results of dried fruit intake on CR level.

(A)Scatter plot of SNPs associated with dried fruit intake on CR level.

(B) Funnel plot of SNPs associated with dried fruit intake on CR level.

(C) Forest plot of SNPs associated with dried fruit intake on CR level.

(D) Leave-one-out analysis of SNPs associated with dried fruit intake on CR level.

Abbreviations: CR, Creatinine; **SNP,** Single nucleotide polymorphism

.

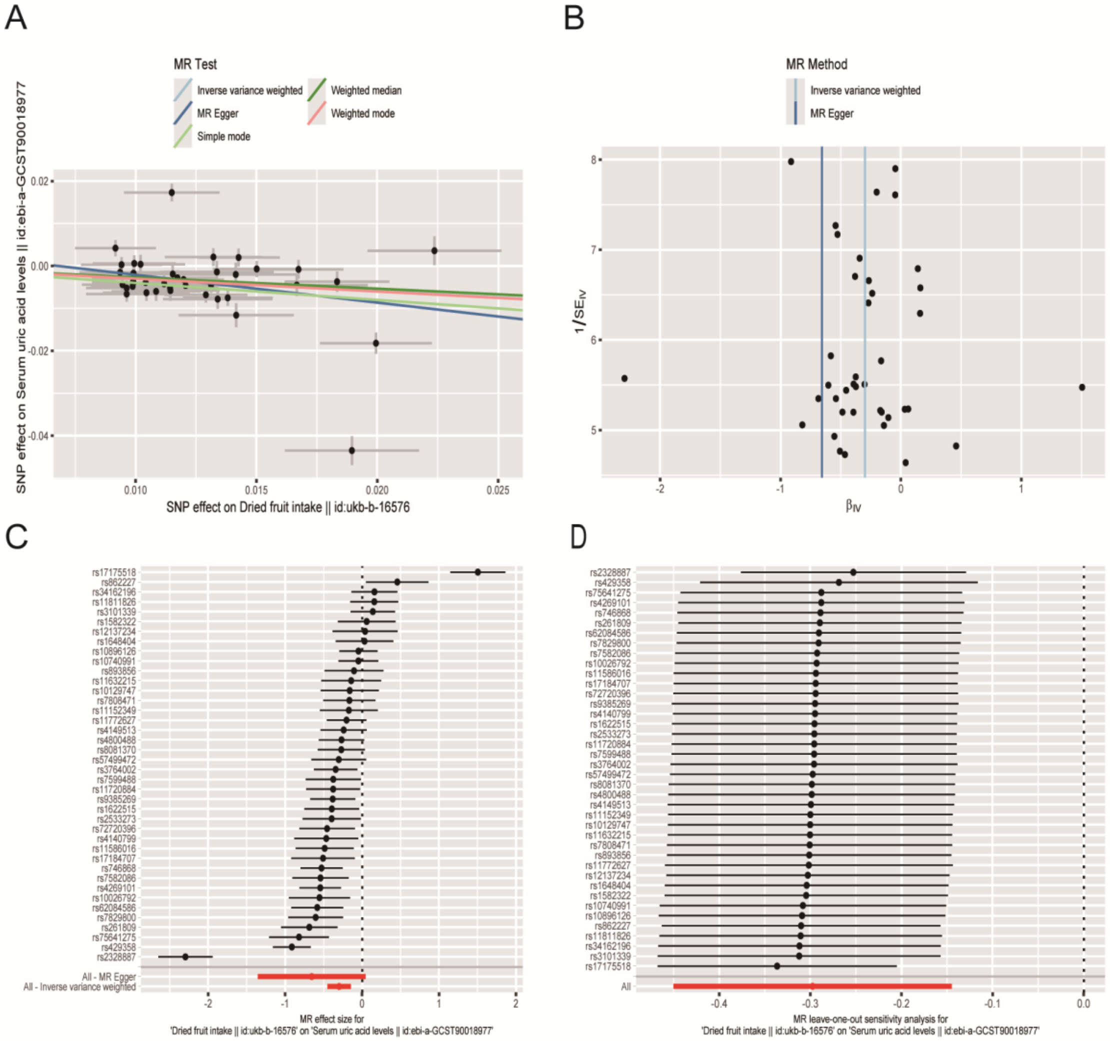

Supplementary Figure 4. MR results of dried fruit intake on UA.

(A) Scatter plot of SNPs associated with dried fruit intake on UA.

(B) Funnel plot of SNPs associated with dried fruit intake on UA.

(C) Forest plot of SNPs associated with dried fruit intake on UA.

(D) Leave-one-out analysis of SNPs associated with dried fruit intake on UA.

Abbreviations: UA, Uric acid**; SNP,** Single nucleotide polymorphism

.

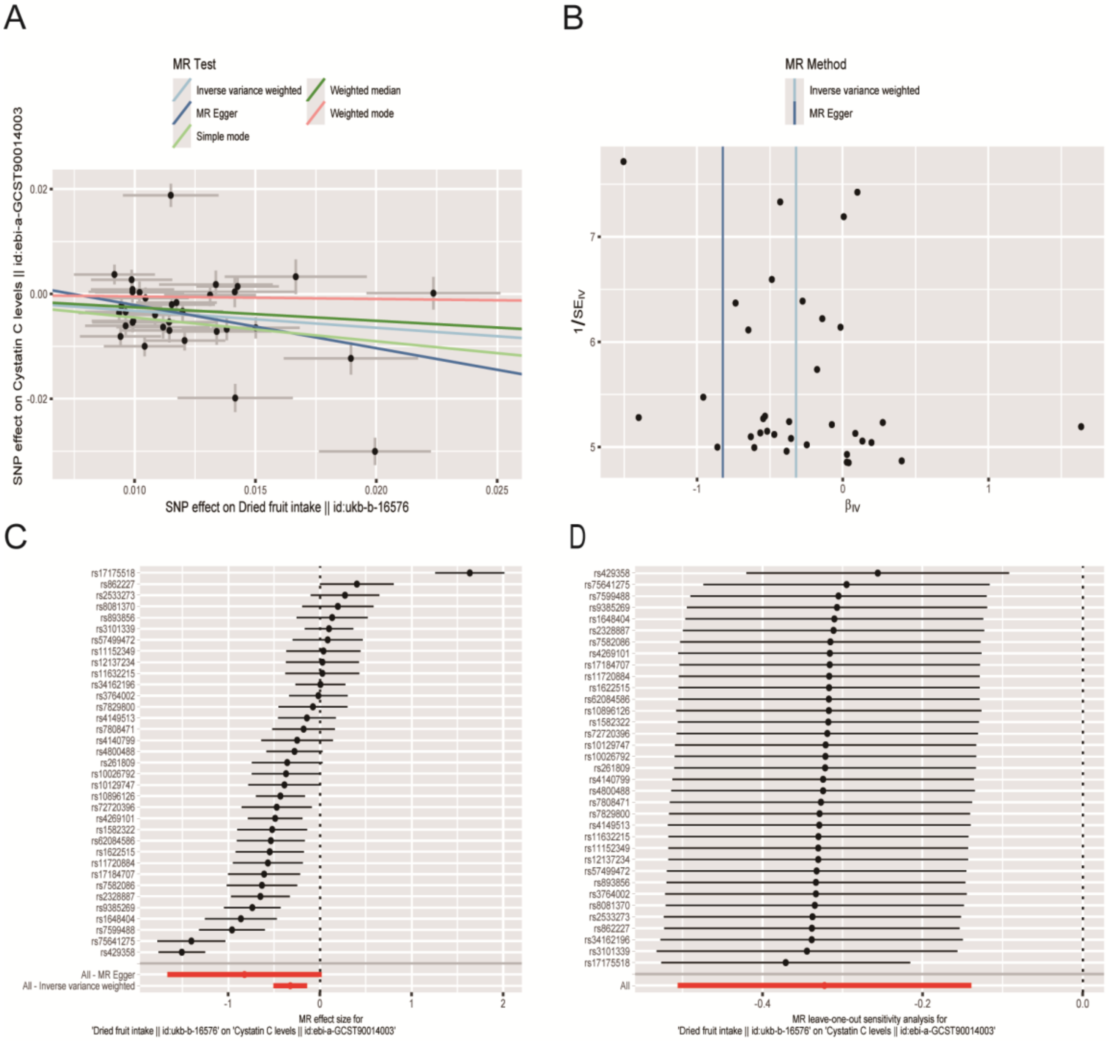

Supplementary Figure 5. MR results of dried fruit intake on CysC.

(A) Scatter plot of SNPs associated with dried fruit intake on CysC.

(B) Funnel plot of SNPs associated with dried fruit intake on CysC.

(C) Forest plot of SNPs associated with dried fruit intake on CysC.

(D) Leave-one-out analysis of SNPs associated with dried fruit intake on CysC.

Abbreviations: CysC, Cystatin C; **SNP,** Single nucleotide polymorphism

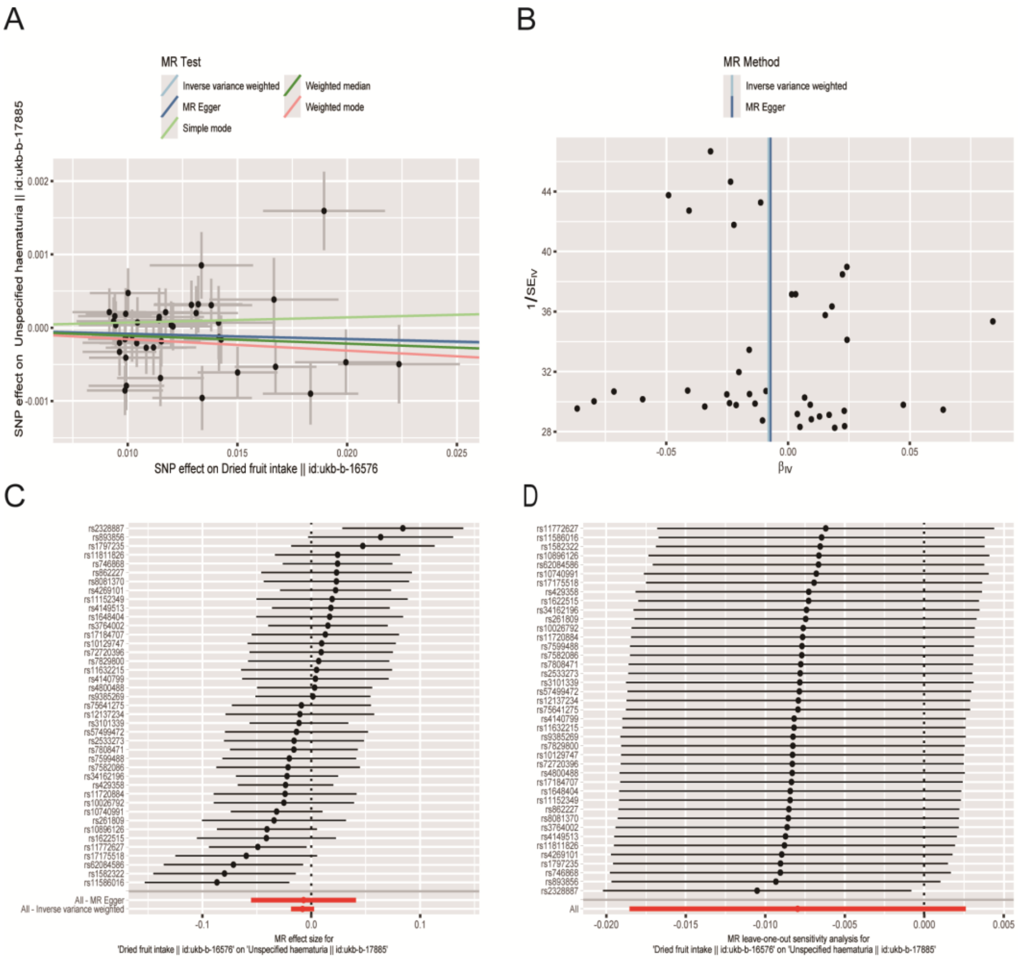

Supplementary Figure 6. MR results of dried fruit intake on hematuria.

(A) Scatter plot of SNPs associated with dried fruit intake on Hematuria

(B) Funnel plot of SNPs associated with dried fruit intake on Hematuria

(C) Forest plot of SNPs associated with dried fruit intake on Hematuria

(D) Leave-one-out analysis of SNPs associated with dried fruit intake on Hematuria.

Abbreviations: Hematuria, Diagnoses - main ICD10: R31 Unspecified hematuria**; SNP,** Single nucleotide polymorphism

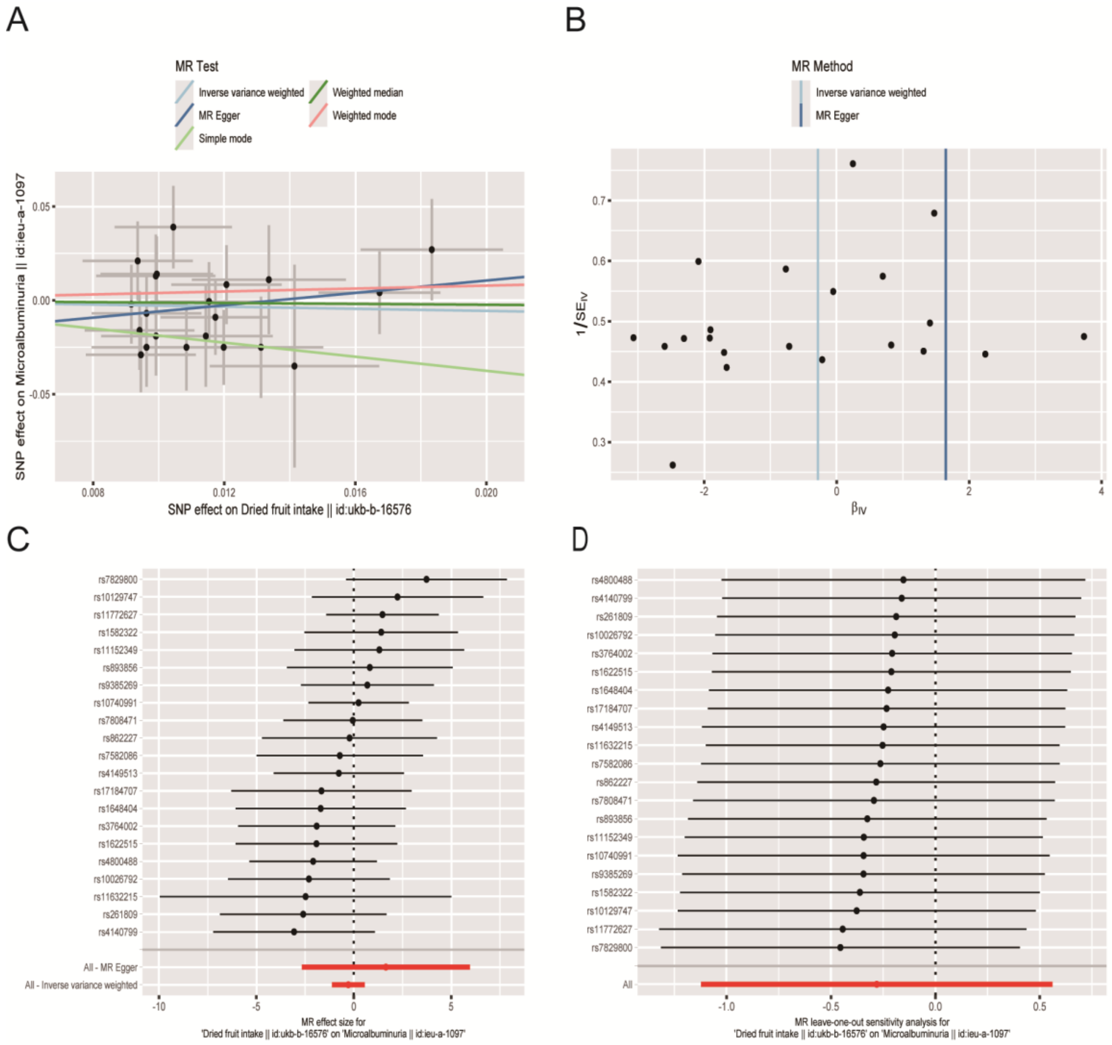

Supplementary Figure 7. MR results of dried fruit intake on MAU.

(A) Scatter plot of SNPs associated with dried fruit intake on MAU.

(B) Funnel plot of SNPs associated with dried fruit intake on MAU.

(C) Forest plot of SNPs associated with dried fruit intake on MAU.

(D) Leave-one-out analysis of SNPs associated with dried fruit intake on MAU.

Abbreviations: MAU, Microalbuminuria; **SNP,** Single nucleotide polymorphism
